# Transcriptomic Signatures of Exercise-Modality Responses in Aged Human Skeletal Muscle

**DOI:** 10.3390/genes17070803

**Published:** 2026-07-15

**Authors:** Sen Yang, Jian Li

**Affiliations:** 1 Institute for Sport Performance and Health Promotion, Capital University of Physical Education and Sports, Beijing 100191, China; 2 Sports & Medicine Integrative Innovation Center, Capital University of Physical Education and Sports, Beijing 100191, China; lijian7593@163.com

**Keywords:** aging, skeletal muscle, exercise training, resistance training, high-intensity interval training, transcriptomics

## Abstract

**Objective:** Exercise training helps preserve skeletal muscle health during aging. However, the molecular responses to different exercise modalities in older adults remain unclear. This study reanalyzed human skeletal muscle transcriptomes to compare signatures associated with combined training, resistance training, and high-intensity interval training. **Method:** We analyzed the older adult subset of GSE97084. This subset included 46 skeletal muscle RNA-seq samples from 23 participants with paired biopsies before and after training. The dataset included seven paired participants in the combined group, eight in the resistance training (RT) group, and eight in the high-intensity interval training (HIIT) group. We performed paired differential expression analysis, GO/KEGG enrichment analysis, GSEA, WGCNA, PPI analysis, regulatory network analysis, and transcriptome-inferred microenvironment signature analysis. **Results:** The within-modality paired comparisons identified 264 DEGs in the combined group, 297 DEGs in the RT group, and 1098 DEGs in the HIIT group. A total of 62 DEGs were shared across all three modalities. Combined training was mainly linked to extracellular matrix (ECM) organization, vascular regulation, and mitochondrial oxidative metabolism. RT showed prominent collagen, ECM, integrin, focal adhesion, and structural remodeling signatures. HIIT showed the broadest DEG profile under the current threshold. HIIT was characterized by vascular endothelial, angiogenic, ECM/adhesion, oxidative phosphorylation, and immune-related microenvironment signatures. WGCNA and PPI analyses identified candidate hub gene patterns. ECM and basement membrane genes were more prominent after combined training and RT. Vascular endothelial genes were more evident after HIIT. Regulatory network analysis highlighted miR-29 family members as database-supported candidate regulators of ECM-related hub genes. Transcriptome-inferred microenvironment analysis suggested increased endothelial-related signatures across all modalities. This analysis also suggested increased fibroblast/stromal signatures after RT and HIIT and increased macrophage-related signatures after HIIT. **Conclusions:** Different exercise modalities were associated with partially overlapping but distinct transcriptomic signatures in aged human skeletal muscle. Combined training and RT were mainly related to ECM, stromal, and structural remodeling signatures. HIIT showed broader vascular endothelial and microenvironment-related signatures. These findings should be interpreted as exploratory because this reanalysis used a modest older adult subset from a single public bulk RNA-seq dataset and lacked an independent validation cohort. Larger studies and complementary experimental validation are needed before drawing definitive conclusions about exercise-modality-specific responses in aged human skeletal muscle.

## 1. Introduction

Aging is linked to a gradual loss of skeletal muscle mass, strength, and metabolic function. These changes increase the risk of poor mobility, frailty, and chronic disease. Sarcopenia is now recognized as a muscle disease that develops across the life course, with low muscle strength as its main clinical feature, followed by reduced muscle mass and impaired physical performance [1]. Aged skeletal muscle also shows mitochondrial dysfunction, impaired regeneration, altered metabolism, and abnormal tissue remodeling [2,3,4]. Exercise training is one of the most effective strategies for preserving muscle function in older adults. Different exercise modalities may produce different molecular responses. Resistance training (RT) improves muscle strength and physical function [5], while high-intensity interval training (HIIT) improves cardiorespiratory fitness, metabolic health, and mitochondrial function [6,7]. Combined training may provide both aerobic and resistance stimuli, but its molecular features remain less clear. Robinson et al. showed that HIIT, RT, and combined training produced distinct physiological and transcriptomic responses in older adults [8]. The RNA-seq data from that study are available as GSE97084, which provides a useful resource for studying exercise-modality-associated signatures in aged human skeletal muscle.

Previous transcriptomic studies have linked exercise training to metabolic, structural, and regulatory pathways in skeletal muscle [8,9]. Multi-omic studies also suggest that exercise adaptation involves coordinated changes across several biological layers [10]. However, many RNA-seq studies still focus mainly on differential expression and pathway enrichment. These approaches are useful, but they may miss coordinated gene modules, candidate regulatory patterns, and tissue microenvironment signatures. This issue is important in aged skeletal muscle because bulk RNA-seq signals may reflect changes in myofibers, as well as endothelial, stromal, immune, satellite/myogenic, and extracellular matrix (ECM)-related components. ECM remodeling is closely linked to skeletal muscle aging and adaptation. The ECM supports force transmission, tissue repair, satellite cell regulation, and metabolic signaling. In aged muscle, altered ECM turnover and collagen deposition may impair muscle quality and regeneration [11]. Exercise training can also reshape ECM-related transcripts and proteins [12]. Vascular adaptation is another key part of skeletal muscle plasticity. Exercise-induced angiogenesis may improve oxygen delivery, metabolite exchange, fatigue resistance, and metabolic health [13]. ECM and vascular remodeling are also connected because matrix composition and stiffness can influence endothelial signaling and angiogenesis [14]. Despite these findings, direct comparisons of HIIT, RT, and combined training remain limited at the transcriptomic systems level. It is still unclear how these exercise modalities relate to ECM, vascular, endothelial, and microenvironment signatures in aged human skeletal muscle.

In this study, we reanalyzed the older adult subset of GSE97084 to characterize transcriptomic signatures associated with combined training, RT, and HIIT. We used paired differential expression analysis and functional enrichment analysis to identify exercise-responsive genes and pathways [15,16]. We used gene set enrichment analysis to examine directional pathway signatures across the transcriptome [17]. We used weighted gene coexpression network analysis to identify training-associated gene modules [18]. We then integrated DEGs with exercise-responsive WGCNA modules to identify candidate hub genes. These genes were further examined using PPI networks, expression confirmation, and coexpression analysis. We also explored candidate TF–mRNA and miRNA–mRNA regulatory relationships using TRRUST and miRTarBase v9.0 through NetworkAnalyst [19,20,21]. Finally, we inferred marker-based microenvironment signatures from bulk RNA-seq data to examine endothelial, stromal, and immune-related patterns [22]. We expected RT and combined training to be more closely linked to ECM, collagen, and structural remodeling signatures, while HIIT would show broader vascular, endothelial, metabolic, and microenvironment-related signatures. This reanalysis provides an exploratory systems-level view of exercise modality-associated transcriptomic patterns in aged human skeletal muscle. Because the older adult subset had a modest sample size and no independent validation cohort, these analyses were used to generate biologically plausible hypotheses rather than to establish definitive modality-specific mechanisms.

## 2. Materials and Methods

### 2.1. Data Source and Study Design

The publicly available skeletal muscle transcriptomic dataset GSE97084 was obtained from the Gene Expression Omnibus (GEO) database of the National Center for Biotechnology Information (NCBI, Bethesda, MD, USA). The raw gene count matrix and matched sample metadata were used for this reanalysis. The original prospective exercise training study included healthy sedentary young adults aged 18–30 years and older adults aged 65–80 years. Participants were assigned to high-intensity interval training (HIIT), resistance training (RT), or combined training, and all interventions lasted 12 weeks. Skeletal muscle biopsy samples were collected before and after training and analyzed as matched pairs. The present reanalysis focused only on the older adult exercise subset. Samples were included if they were from older adults, derived from skeletal muscle biopsy tissue, belonged to one of the three exercise modalities, and had complete metadata for participant identity, the exercise modality, and the time point. Participants without matched pre- and post-training samples were excluded, as were younger participants, samples from other training conditions, and samples with incomplete metadata.

After filtering and metadata matching, 46 skeletal muscle samples from 23 older adults were retained for downstream analysis. These samples included 7 paired participants in the combined group, 8 paired participants in the RT group, and 8 paired participants in the HIIT group. The paired design was maintained throughout the analysis to reduce the influence of inter-individual baseline differences. The present reanalysis did not include a younger reference cohort, so age-dependent differences in exercise-induced transcriptomic responses could not be directly assessed. Because this study used bulk RNA-seq count data, the results were interpreted as mRNA-level transcriptomic evidence rather than direct measurements of protein abundance, histological structure, or cell-type proportions. No independent validation cohort with matched older adult pre- and post-training RNA-seq samples across the same exercise modalities was available. Therefore, downstream network, regulatory, and microenvironment signature analyses were interpreted as exploratory and hypothesis-generating.

### 2.2. Data Extraction, Preprocessing, and Gene Annotation

The raw count matrix and sample metadata were imported into R software (version 4.5.0, R Foundation for Statistical Computing, Vienna, Austria). The original GSE97084 count data were stored in two Excel files, and target older adult samples were extracted from each file according to the matched metadata. Each count table was formatted using Ensembl gene identifiers and sample count columns. Rows with missing gene identifiers were removed, count values were converted to numeric format, and duplicated gene identifiers within each file were collapsed by summing raw counts. The two count tables were then merged by gene identifier, and missing values introduced during merging were set to zero. The final count matrix was reordered to match the sample order in the metadata table, and sample names were checked for consistency with the metadata. Metadata variables, including the participant identity, age group, exercise modality, and time point, were converted to factor format. Low-expression genes were removed before differential expression and downstream analyses, retaining genes with raw counts ≥10 in at least two samples. For gene annotation, Ensembl gene identifiers were cleaned by removing version suffixes, and official gene symbols were annotated using AnnotationDbi::mapIds with org.Hs.eg.db. Genes without mapped symbols retained their original gene identifiers, and duplicated display labels were made unique only for visualization.

### 2.3. Quality Control and Variance-Stabilizing Transformation

Raw count distributions were examined after low-expression gene filtering. Raw counts were transformed as log10(raw count + 1) and visualized using boxplots across samples to assess expression ranges and possible sample-level distribution differences. Variance-stabilizing transformation was performed using the DESeq2 R/Bioconductor package with a DESeqDataSet object constructed from the filtered count matrix and matched metadata. VST was performed with blind = TRUE because the transformed matrix was used for quality control and exploratory visualization. The VST-transformed expression matrix was then used for PCA and heatmap visualization. The distribution of VST-transformed values was examined to assess cross-sample comparability after transformation. PCA was performed on the transposed VST matrix using prcomp() without additional scaling. The first two principal components were used to show the overall transcriptomic structure of older adult skeletal muscle samples, and paired PCA trajectories were generated by connecting pre- and post-training samples from the same participant within each exercise modality.

### 2.4. Differential Expression Analysis

We performed differential expression analysis separately for the combined, RT, and HIIT groups. In each group, post-training samples were compared with matched pre-training samples. Genes with raw counts ≥10 in at least two samples were retained. Pair completeness was checked before analysis. We used DESeq2 with a paired design. The model included participant identity and the time point: design = ~ subject_id + time. The training effect was estimated by comparing post with pre. *p* values were adjusted using the Benjamini–Hochberg method. Genes with an adjusted *p* value < 0.05 and |log2 fold change| ≥ 0.5 were defined as differentially expressed genes. We also performed an exploratory time-by-modality interaction analysis using all 46 samples from 23 paired participants. This analysis tested whether post-training expression changes differed among the combined, RT, and HIIT groups. Because of the modest sample size, these interaction results were interpreted as exploratory.

Volcano plots were used to show differential expression patterns. Upregulated genes were shown in red, with downregulated genes in blue and non-significant genes in gray. Only selected top-ranked significant genes were labeled to reduce crowding. Heatmaps showed up to 30 top-ranked significant DEGs ranked by adjusted *p* value. Expression values were based on the VST-transformed matrix and scaled by the row z-score. Pre- and post-training samples were annotated in gray and orange, respectively, to avoid confusion with the red and blue colors used in the volcano plots.

### 2.5. Functional Enrichment Analysis of DEGs

Functional enrichment analysis was performed separately for the combined, RT, and HIIT groups. Significant DEGs from the paired differential expression analysis were used for this analysis. Gene identifiers were converted to ENTREZ IDs using org.Hs.eg.db. For each exercise modality, significant DEGs with valid ENTREZ IDs were used as the input gene list. All tested genes with valid ENTREZ IDs in the corresponding DESeq2 comparison were used as the background universe.

GO enrichment analysis was performed using the clusterProfiler R/Bioconductor package. Biological process, cellular component, and molecular function categories were included. KEGG pathway enrichment analysis was performed using the clusterProfiler R/Bioconductor package, with the organism set to *Homo sapiens* (*hsa*). The Benjamini–Hochberg method was used for multiple-testing correction. Terms with an adjusted *p* value < 0.05 were considered significant. Nominally significant terms were also reviewed when they helped show representative biological patterns.

Enrichment results were shown using combined gene–term link plots and dot plots. For each exercise modality, representative GO terms and KEGG pathways were selected according to the enrichment significance and biological relevance. Highly redundant terms with overlapping gene sets were reduced to make the figure easier to read. In the gene–term link plots, DEGs were connected to selected terms or pathways. In the dot plots, the x-axis showed the GeneRatio. The dot size showed the number of DEGs assigned to each term. The dot color showed enrichment significance as −log10(*p* value). The number of displayed terms and genes was limited to improve readability. Disease- or organ-labeled terms were interpreted according to their contributing genes and biological functions. They were not interpreted as direct evidence of disease- or organ-specific changes.

### 2.6. Gene Set Enrichment Analysis

Gene set enrichment analysis was performed separately for the combined, RT, and HIIT groups. For each modality, genes from the paired DESeq2 comparison were ranked mainly by the DESeq2 Wald statistic. Gene identifiers were converted to ENTREZ IDs, and duplicated ENTREZ IDs were resolved by retaining the entry with the largest absolute ranking score. GO GSEA was performed using the clusterProfiler R/Bioconductor package, including biological process, cellular component, and molecular function categories. KEGG GSEA was performed using Molecular Signatures Database (MSigDB) KEGG_MEDICUS or KEGG gene sets when available; otherwise, clusterProfiler::gseKEGG was used. Gene set sizes were limited to 10–500 genes. The Benjamini–Hochberg method was used for multiple-testing correction. The normalized enrichment score (NES) was used to indicate the enrichment direction. Positive NES values indicated enrichment among genes relatively upregulated after training, whereas negative NES values indicated enrichment among genes relatively downregulated after training. GSEA results were shown as NES dot plots. The dot size represented the number of core enriched genes, and the dot color represented the adjusted *p* value. Only representative top-ranked pathways were shown in the main figure to improve readability. Disease-related pathway names were interpreted according to their contributing genes rather than as direct evidence of disease-specific changes.

### 2.7. Weighted Gene Co-Expression Network Analysis

Weighted gene coexpression network analysis (WGCNA) was performed using the WGCNA R package to identify exercise-responsive coexpression modules. The analysis used paired skeletal muscle samples collected before and after training from the combined, RT, and HIIT groups. Low expression genes were removed before network construction. The top 5000 most variable genes were used for WGCNA. The soft thresholding power was selected using the scale-free topology criterion. A power of β = 8 was used for network construction. Coexpression modules were identified using hierarchical clustering and dynamic tree cutting. Module eigengenes were calculated to summarize module-level expression patterns. Changes in module eigengenes after training were then compared across the combined, RT, and HIIT groups. Effect sizes and *p* values were estimated for each module and exercise modality. *p* values were adjusted using the Benjamini–Hochberg method. Global FDR correction across all module and training tests and within-group FDR correction were both calculated. Modules with FDR adjusted *p* values < 0.05 were considered significant. WGCNA results were visualized using soft threshold diagnostic plots, a gene clustering dendrogram with module colors, and a heatmap of module eigengene changes after training. The heatmap showed effect sizes and nominal *p* values for each module and exercise modality. WGCNA was interpreted as an exploratory coexpression analysis because no independent validation cohort was available.

### 2.8. Candidate Core Gene Screening and PPI Network Construction

Candidate core genes were identified by intersecting significant DEGs with genes from exercise-responsive WGCNA modules. This analysis was performed separately for the combined, RT, and HIIT groups. Shared and modality-related candidate genes were summarized using a Venn diagram. PPI networks were constructed for each candidate gene set using STRING database-based interaction data, with *H. sapiens* as the reference organism. The default STRING confidence setting was used. Only candidate genes from the corresponding exercise modality were retained. Duplicate interaction pairs were collapsed when present. PPI networks were treated as undirected graphs because the interactions represented protein associations rather than directional regulatory relationships. Network analysis was performed using the igraph R package in R. Degree centrality was calculated for each node and used to rank candidate hub genes. The selected hub genes were then used for expression confirmation, coexpression analysis, and TF, mRNA, and miRNA regulatory network analysis.

### 2.9. Core Gene Expression Confirmation and Co-Expression Analysis

Normalized expression values of selected hub genes were extracted from the processed expression matrix and expressed as log2(CPM + 1). Within each exercise modality, pre-training and post-training expression levels were compared using paired Wilcoxon signed-rank tests. Violin plots with overlaid boxplots and paired sample lines were used to show expression changes after training. This analysis was interpreted as expression confirmation rather than independent validation because the selected hub genes were identified from the same dataset through the DEG, WGCNA, and PPI workflow. Spearman correlation analysis was used to assess coordinated expression patterns among selected hub genes based on post-training minus pre-training expression changes.

### 2.10. TF–mRNA–miRNA Regulatory Network Analysis

TF and miRNA regulatory relationships were analyzed to explore possible upstream regulators of selected hub genes. TF–mRNA interactions were obtained from the TRRUST database through the NetworkAnalyst web platform (Xia Lab, McGill University, Montreal, QC, Canada), and miRNA–mRNA interactions were obtained from miRTarBase v9.0 through NetworkAnalyst. The analysis was performed separately for the combined, RT, and HIIT groups. Selected hub genes from each exercise modality were used as input targets, and only regulatory edges connected to these hub genes were retained. The regulator degree was defined as the number of connected hub genes. Shared and modality-related TFs and miRNAs were summarized using presence plots. An alluvial plot was used to summarize the relationships among exercise modality, regulator, hub gene, and functional category, including basement membrane, ECM remodeling, collagen organization, angiogenesis, endothelial junction, and vascular remodeling. This analysis was interpreted as exploratory because all regulatory relationships were derived from public databases. Therefore, these results suggest possible TF and miRNA regulatory relationships, but they do not provide direct evidence of regulator activity or causal regulation in the present dataset.

### 2.11. Transcriptome-Inferred Skeletal Muscle Microenvironment Analysis

These signatures represented endothelial, pericyte/mural, fibroblast/stromal, ECM/collagen, macrophage, T cell, B cell, satellite/myogenic, and adipocyte-related patterns. Only marker genes detected in the expression matrix were used for the score calculation. Raw counts were normalized to log2(CPM + 1), and marker gene expression values were standardized across samples using gene-wise z-score transformation. For each signature, the mean z-score of available marker genes was calculated as the signature score for each sample. Paired comparisons before and after training were performed separately in the combined, RT, and HIIT groups using paired Wilcoxon signed-rank tests. Endothelial, fibroblast/stromal, and macrophage signatures were selected for paired visualization. Heatmaps were used to show overall transcriptome-inferred microenvironment patterns, and violin plots with overlaid boxplots and paired sample lines were used to show changes in selected signatures after training. Post-training minus pre-training changes were calculated for microenvironment signatures and hub module scores. Spearman correlation analysis was used to assess relationships between delta microenvironment scores and delta hub module scores, and subject-level delta-response analysis was used to compare response magnitudes among exercise modalities. These analyses were interpreted as transcriptome-inferred signature analyses rather than direct evidence of changes in cell-type abundance.

### 2.12. Statistical Analysis and Visualization

All statistical analyses and visualizations were performed in R 4.5.0. Differential expression analysis and exploratory gene-level time-by-modality interaction analysis were performed using DESeq2. The overall interaction was tested using a likelihood ratio test, and pairwise interaction contrasts were tested using Wald tests. Functional enrichment analysis and GSEA were performed using clusterProfiler and related Bioconductor annotation packages. Co-expression network analysis was performed using WGCNA. Network analysis and figure generation were performed using ggplot2, pheatmap, patchwork, igraph, ggraph, tidyverse, and openxlsx. Package versions and session information were recorded using sessionInfo(). For non-DESeq2 paired comparisons, pre-training and post-training values were compared using paired Wilcoxon signed-rank tests. Spearman correlation analysis was used to assess associations among gene expression changes, module scores, hub gene signatures, and microenvironment scores. All statistical tests were two-sided.

For analyses involving multiple testing, *p* values were adjusted using the Benjamini–Hochberg method. Differential expression results were evaluated using adjusted *p* values. GO/KEGG enrichment analysis, GSEA, interaction tests, and delta-response analyses also used adjusted *p* values when multiple comparisons were performed. Nominal *p* values < 0.05 were reported only as exploratory findings when FDR-adjusted results were not significant. WGCNA, candidate hub gene prioritization, PPI network analysis, regulatory network analysis, and transcriptome-inferred microenvironment analysis were considered exploratory and were used to identify candidate modules, hub genes, regulatory relationships, and marker-based transcriptomic signatures. A subject-level delta-response analysis was performed to examine whether response magnitudes differed among exercise modalities. Delta response was calculated as the post-training score minus pre-training score. Delta scores were compared among the combined, RT, and HIIT groups using Kruskal–Wallis tests, followed by pairwise Wilcoxon rank-sum tests when appropriate. Cliff’s delta was calculated to estimate pairwise effect sizes.

## 3. Results

### 3.1. Overview and Quality Control of the Aged Skeletal Muscle Transcriptomic Dataset

**Figure 1:**
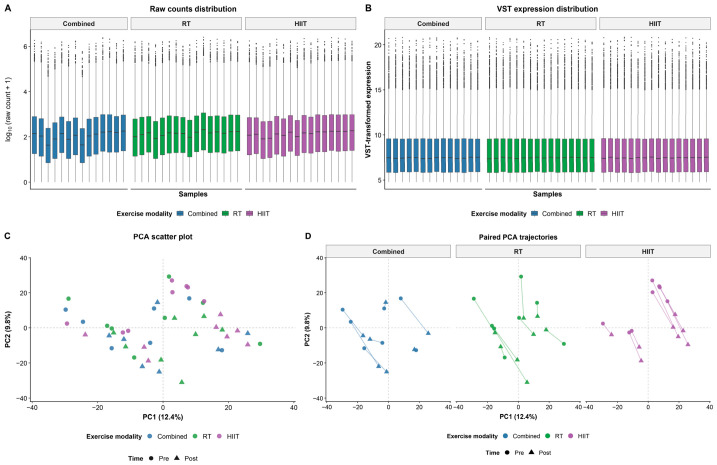
Dataset overview and quality control of older adult skeletal muscle RNA-seq samples. (**A**) Raw count values across all retained samples, shown on a log10 scale. (**B**) Variance-stabilizing transformation (VST)-transformed expression values across all retained samples. (**C**) Principal component analysis (PCA) plot based on the VST-transformed expression matrix. Colors indicate the exercise modality, and shapes indicate the time point. (**D**) Paired PCA trajectory plot showing pre- to post-training changes for each participant within each exercise modality.

The raw count data showed a broad dynamic range, which is typical of bulk RNA-seq datasets. On the log10 scale, the overall expression distributions were broadly comparable across samples, although some differences remained in the box height, whisker length, and outlier density (Figure 1A). These patterns suggested that residual expression heterogeneity was present in the raw count matrix. They also supported the need for normalization and variance-stabilizing transformation before exploratory downstream analyses. After VST transformation, the expression distributions became more consistent across samples. The differences in box height and whisker length were reduced, and the expression ranges became more comparable across the combined, RT, and HIIT groups (Figure 1B). These results indicate that VST reduced the mean–variance dependence of count-based RNA-seq data and improved cross-sample comparability. Therefore, the VST-transformed expression matrix was used for PCA, heatmap visualization, and co-expression network analysis. The final analysis included 46 skeletal muscle biopsy samples from older adults, representing 23 matched pre- and post-training pairs. The dataset included seven pairs in the combined group, eight pairs in the RT group, and eight pairs in the HIIT group. This paired design helped control inter-individual baseline differences in the differential expression analyses. We then used PCA to examine the overall transcriptomic structure of the aged skeletal muscle samples based on the VST-transformed expression matrix. No severe outlier was detected after preprocessing, and all samples remained within the main PCA distribution (Figure 1C). Samples from the three exercise modalities partially overlapped in PCA space. This pattern suggests that global transcriptomic variation was not driven only by the exercise modality or time point. The paired PCA trajectory plot showed detectable within-subject shifts from pre- to post-training samples (Figure 1D). The direction and magnitude of these shifts varied across participants. However, paired trajectories were observed within each exercise modality. These results suggest that exercise training was associated with measurable but heterogeneous transcriptomic changes in aged skeletal muscle.

### 3.2. Exercise Training Is Associated with Modality-Related Differential Expression Patterns

**Figure 2:**
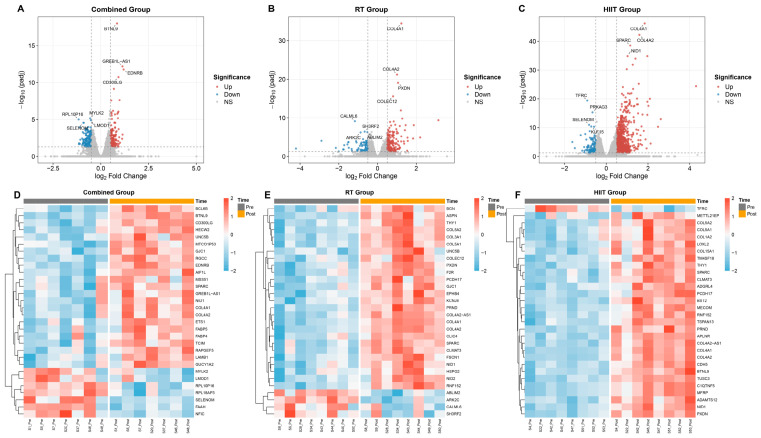
Differential expression patterns associated with exercise training in older adults. (**A**) Volcano plot showing differentially expressed genes in the combined group based on the paired post-training versus pre-training comparison. (**B**) Volcano plot showing differentially expressed genes in the RT group. (**C**) Volcano plot showing differentially expressed genes in the HIIT group. (**D**) Heatmap showing up to 30 top-ranked DEGs in the combined group, ranked by adjusted *p* value. (**E**) Heatmap showing up to 30 top-ranked DEGs in the RT group. (**F**) Heatmap showing up to 30 top-ranked DEGs in the HIIT group. In the volcano plots, upregulated genes are shown in red, downregulated genes are shown in blue, and non-significant genes are shown in gray. The dotted vertical lines indicate the log2 fold change cutoffs of −0.5 and 0.5, and the dotted horizontal line indicates the adjusted p value cutoff of 0.05. Only selected representative top-ranked genes are labeled to reduce crowding. Heatmaps are based on VST-transformed expression values scaled by row z-score. Pre- and post-training samples in the heatmaps are indicated by gray and orange annotations, respectively, to avoid confusion with the red and blue colors used for DEG directionality in the volcano plots. DEGs were identified from within-modality paired post-training versus pre-training comparisons.

Paired differential expression analysis was performed separately in the combined, RT, and HIIT groups. Each analysis compared post-training samples with matched pre-training samples. Genes with an adjusted *p* value < 0.05 and an absolute log2 fold change ≥ 0.5 were defined as differentially expressed genes (DEGs). Volcano plots were used to show the overall differential expression pattern in each exercise modality. Heatmaps were used to show the expression patterns of representative top-ranked DEGs. To improve readability, each volcano plot labeled only selected top-ranked significant genes. Each heatmap displayed up to 30 top-ranked DEGs. The heatmaps used gray and orange annotations for pre- and post-training samples. This color scheme avoided confusion with the red and blue colors used for upregulated and downregulated genes in the volcano plots. In the combined group, 264 DEGs were identified after training (Figure 2A,D). In the RT group, 297 DEGs were identified after training (Figure 2B,E). The HIIT group showed the largest DEG profile under the current statistical threshold, with 1098 DEGs identified after training (Figure 2C,F). The heatmaps showed clear or relatively consistent expression shifts between pre- and post-training samples across the three exercise modalities. These results suggest that HIIT was associated with the broadest transcriptional response in aged skeletal muscle under the current threshold. All comparisons were performed within each exercise modality. Therefore, these findings should be interpreted as modality-related response patterns, not as definitive evidence of statistically confirmed between-modality differences.

Supplementary visualizations were generated to summarize DEG numbers and overlaps across exercise modalities. The DEG number comparison is shown in Supplementary Figure S1A, and the DEG overlap among the three exercise modalities is shown in Supplementary Figure S1B. The combined, RT, and HIIT groups contained 264, 297, and 1098 DEGs, respectively. A total of 62 DEGs were shared across all three groups. This shared set may represent a common exercise-responsive gene signature in aged skeletal muscle. In addition, 127 DEGs were unique to the combined group, 98 were unique to the RT group, and 844 were unique to the HIIT group. Pairwise overlaps were also observed. Ten DEGs were shared only between the combined and RT groups. Sixty-five DEGs were shared only between the combined and HIIT groups. One hundred twenty-seven DEGs were shared only between the RT and HIIT groups. We further tested whether post-training transcriptional responses differed among exercise modalities using an exploratory gene-level time-by-modality interaction analysis. The overall likelihood ratio test identified 1660 genes with nominal evidence of interaction at *p* < 0.05, but no gene remained significant after Benjamini–Hochberg correction at FDR < 0.05. Pairwise Wald interaction contrasts also did not identify any FDR-significant genes. These results indicate that the present dataset did not provide robust FDR-supported evidence for gene-level time-by-modality interactions. Therefore, the differences observed among exercise modalities should be interpreted as exploratory modality-associated patterns.

### 3.3. Functional Enrichment Analysis Reveals Modality-Associated Biological Signatures After Exercise Training

**Figure 3:**
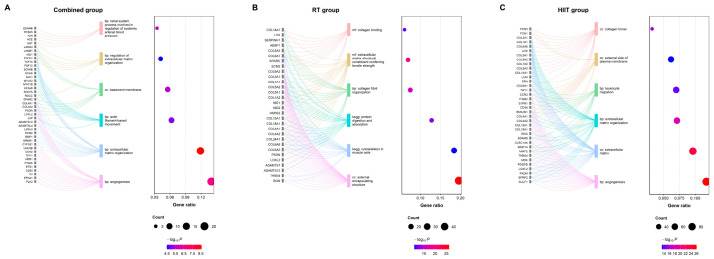
Functional enrichment analysis of exercise-responsive differentially expressed genes in older skeletal muscle. (**A**) Representative GO/KEGG enrichment results for DEGs in the combined group. (**B**) Representative GO/KEGG enrichment results for DEGs in the RT group. (**C**) Representative GO/KEGG enrichment results for DEGs in the HIIT group. Each panel includes a gene–term link plot and a dot plot. In the gene–term link plot, DEGs are connected to selected enriched GO terms or KEGG pathways. In the dot plot, the x-axis represents the GeneRatio, the dot size indicates the number of DEGs assigned to each term, and the color represents enrichment significance as −log10(*p* value). The combined group was mainly enriched in extracellular matrix organization, basement membrane, angiogenesis, and vascular regulation-related terms. The RT group was mainly associated with collagen binding, collagen fibril organization, extracellular matrix structural components, and muscle cytoskeleton-related pathways. The HIIT group was enriched in collagen trimer, extracellular matrix organization, angiogenesis, leukocyte migration, and plasma membrane-related terms.

We performed GO and KEGG enrichment analyses to characterize the biological processes associated with exercise-responsive DEGs. Each exercise modality was analyzed separately. Figure 3 summarizes representative enriched terms for the combined, RT, and HIIT groups. Each panel links selected DEGs to enriched terms and shows the corresponding GeneRatio, gene count, and enrichment significance.

In the combined group, DEGs were mainly enriched in extracellular matrix and vascular remodeling-related terms (Figure 3A). Representative terms included regulation of extracellular matrix organization, basement membrane, extracellular matrix organization, angiogenesis, and actin filament-based movement. These terms involved several ECM- and basement membrane-related genes, such as *LAMA4*, *LAMB1*, *NID1*, *COL4A1*, *COL4A2*, *PXDN*, *LOXL2*, *LOXL4*, *SPARC*, and *BMP1*. A term related to renal system regulation of systemic arterial blood pressure was also enriched. We interpreted this term according to its vascular-regulatory genes rather than as evidence of renal functional changes. These results suggest that combined training was associated with ECM remodeling, basement membrane structure, angiogenesis, and vascular regulatory signatures in aged skeletal muscle. In the RT group, DEGs were mainly enriched in collagen and extracellular matrix-related terms (Figure 3B). Representative terms included collagen binding, extracellular matrix structural constituent conferring tensile strength, collagen fibril organization, cytoskeleton in muscle cells, external encapsulating structure, and protein digestion and absorption. These annotations were largely driven by collagen and ECM-related genes, including *COL1A1*, *COL1A2*, *COL3A1*, *COL5A1*, *COL5A2*, *COL5A3*, *COL6A1*, *COL6A2*, *COL14A1*, *NID1*, *HSPG2*, *PXDN*, *LOXL2*, and *BMP1*. The enrichment of cytoskeleton-related terms suggests that RT was associated with extracellular structural remodeling and muscle cytoskeletal signatures. The KEGG term protein digestion and absorption was mainly driven by collagen-related genes. We therefore interpreted this term as part of a broader collagen/ECM annotation pattern, not as evidence of altered digestive function. In the HIIT group, DEGs were enriched in vascular, extracellular matrix, and immune-related terms (Figure 3C). Representative terms included collagen trimer, external side of plasma membrane, leukocyte migration, extracellular matrix organization, extracellular matrix, and angiogenesis. These terms involved collagen genes, endothelial or vascular-related genes, and immune-associated genes. Compared with the combined and RT groups, HIIT showed stronger enrichment for angiogenesis, leukocyte migration, and broader microenvironment-related processes. These results suggest that HIIT was associated with vascular remodeling, ECM remodeling, and immune-related transcriptomic signatures in aged skeletal muscle.

Overall, functional enrichment analysis showed that exercise-responsive DEGs were concentrated in tissue-remodeling-related processes. Combined training was mainly associated with ECM organization, basement membrane structure, and vascular regulation. RT showed a collagen/ECM-centered structural remodeling pattern. HIIT showed broader vascular, ECM, leukocyte migration, and angiogenesis-related signatures. These findings support the presence of modality-associated functional patterns. However, these results should be interpreted cautiously. The enrichment analyses were based on within-modality DEG lists rather than formal gene-level between-modality interaction tests.

### 3.4. GSEA Reveals Directional Pathway-Level Transcriptional Signatures After Exercise Training

We performed GSEA separately in the combined, RT, and HIIT groups. Genes were ranked using statistics from paired post-training versus pre-training comparisons. Positive NES values indicated enrichment among genes that were relatively upregulated after training. Negative NES values indicated enrichment among genes that were relatively downregulated after training. We interpreted these results as directional transcriptomic signatures, not as direct evidence of pathway activation or inhibition.

**Figure 4:**
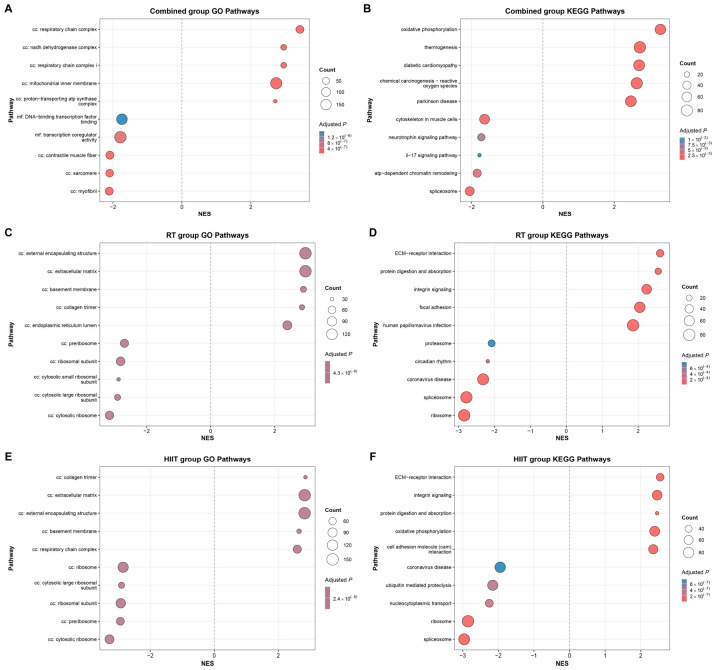
GSEA of GO and KEGG pathways associated with exercise training in older skeletal muscle. (**A**) GO GSEA NES dot plot for the combined group. (**B**) KEGG GSEA NES dot plot for the combined group. (**C**) GO GSEA NES dot plot for the RT group. (**D**) KEGG GSEA NES dot plot for the RT group. (**E**) GO GSEA NES dot plot for the HIIT group. (**F**) KEGG GSEA NES dot plot for the HIIT group. The x-axis shows the normalized enrichment score (NES). Positive NES values indicate enrichment among genes relatively upregulated after training. Negative NES values indicate enrichment among genes relatively downregulated after training. The dot size shows the number of core enriched genes. The dot color shows the adjusted *p* value. Only representative top-ranked pathways are shown to improve readability. These GSEA results were interpreted as directional transcriptomic signatures, not as direct evidence of pathway activation or suppression.

In the combined group, GO GSEA showed the positive enrichment of mitochondrial and respiratory chain terms. These terms included the respiratory chain complex, NADH dehydrogenase complex, mitochondrial inner membrane, and ATP synthase complex (Figure 4A). KEGG GSEA also showed the positive enrichment of oxidative phosphorylation and thermogenesis (Figure 4B). Some disease-labeled pathways, including diabetic cardiomyopathy and Parkinson disease, were mainly driven by mitochondrial respiration genes. We interpreted these pathways as mitochondrial energy metabolism signatures. Negative NES values were observed for contractile muscle fiber, sarcomere, myofibril, cytoskeleton in muscle cells, spliceosome, and chromatin remodeling terms. In the RT group, GO GSEA showed the positive enrichment of extracellular matrix and collagen terms. These terms included the external encapsulating structure, extracellular matrix, basement membrane, collagen trimer, and endoplasmic reticulum lumen (Figure 4C). KEGG GSEA showed the positive enrichment of ECM–receptor interaction, integrin signaling, focal adhesion, and protein digestion and absorption (Figure 4D). The protein digestion and absorption pathway was mainly driven by collagen genes. We interpreted this pathway as part of a broader ECM and collagen remodeling pattern. Negative NES values were mainly found for ribosomal, spliceosome, and proteasome terms. In the HIIT group, GO GSEA showed the positive enrichment of collagen trimer, extracellular matrix, external encapsulating structure, basement membrane, and respiratory chain complex (Figure 4E). KEGG GSEA showed the positive enrichment of ECM–receptor interaction, integrin signaling, oxidative phosphorylation, and cell adhesion molecule interaction (Figure 4F). Negative NES values were observed for ribosome, spliceosome, nucleocytoplasmic transport, and ubiquitin-mediated proteolysis.

Overall, GSEA showed distinct pathway signatures across exercise modalities. Combined training was mainly linked to mitochondrial respiratory chain and oxidative phosphorylation signatures. RT showed a stronger ECM, collagen, and adhesion pattern. HIIT showed both ECM, adhesion, and mitochondrial oxidative metabolism signatures. These findings should be interpreted as transcriptomic pathway patterns. They do not provide direct evidence of protein, metabolic, or physiological pathway activity.

### 3.5. WGCNA Identifies Exercise-Responsive Co-Expression Modules in Aged Skeletal Muscle

**Figure 5:**
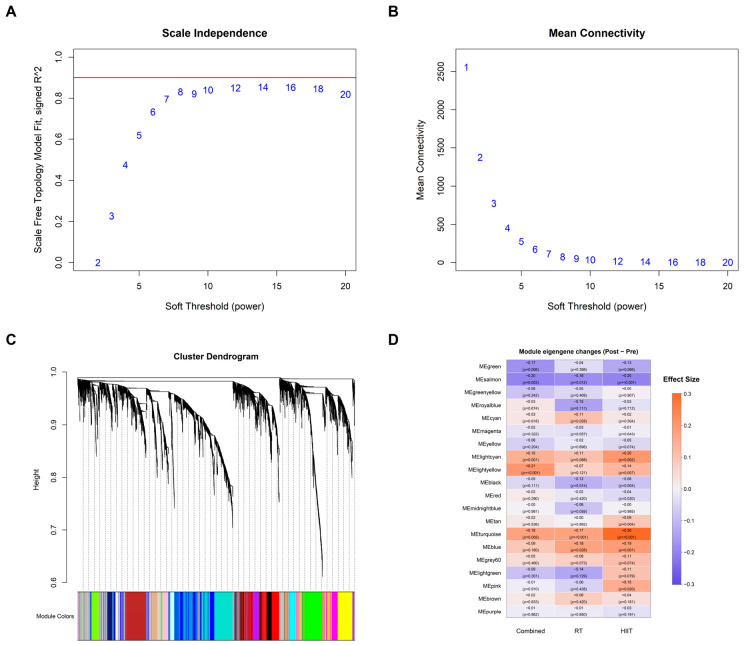
WGCNA characterization of exercise-responsive co-expression modules in older skeletal muscle. (**A**) Scale-free topology model fit across candidate soft-thresholding powers. The red horizontal line shows the reference threshold for scale-free topology fit. A soft-thresholding power of β = 8 was selected for network construction. (**B**) Mean connectivity across candidate soft-thresholding powers. (**C**) Gene clustering dendrogram based on topological overlap dissimilarity. Module colors below the dendrogram show the co-expression modules identified by dynamic tree cutting. (**D**) Heatmap showing post-training minus pre-training changes in module eigengene values across the combined, RT, and HIIT groups. Each cell shows the effect size, with the nominal *p* value shown in parentheses. Color shows the direction and magnitude of the module eigengene change. WGCNA was performed using the top 5000 most variable genes from 46 skeletal muscle samples. Module-level findings were interpreted as exploratory co-expression signatures.

We performed WGCNA to identify coordinated gene expression patterns associated with exercise training in aged skeletal muscle. The analysis used the older-adult exercise subset and focused on module-level changes after training. The scale-free topology fit increased as the soft-thresholding power increased, while mean connectivity decreased (Figure 5A,B). Based on these diagnostic results, we selected a soft-thresholding power of β = 8 for network construction. Genes were then clustered using topological overlap dissimilarity, and co-expression modules were identified from the resulting dendrogram (Figure 5C). Module eigengenes were calculated to summarize module-level expression patterns, and post-training minus pre-training module eigengene changes were compared across the combined, RT, and HIIT groups (Figure 5D).

In the combined group, MElightcyan, MElightyellow, and MEturquoise showed positive module-level changes, whereas MEgreen and MEsalmon showed negative changes. In the RT group, MEturquoise showed the strongest positive change, whereas MEsalmon showed a negative change, suggesting a more selective module-level response pattern. In the HIIT group, several modules showed clear module-level changes. MEturquoise showed the largest positive change among the tested module-training pairs and remained significant after FDR correction. MEblue, MElightcyan, MEtan, and MElightyellow also showed positive changes after HIIT, whereas MEsalmon and MEblack showed negative changes.

Across exercise modalities, MEturquoise showed positive changes in the combined, RT, and HIIT groups, suggesting that this module may represent a shared exercise-responsive co-expression signature in aged skeletal muscle. In contrast, MEsalmon showed negative changes across all three groups, suggesting a commonly downregulated module-level response after training. Overall, WGCNA identified coordinated co-expression modules associated with exercise training. HIIT showed the broadest module-level response, combined training also showed multiple module-level changes, and RT showed a more selective pattern. Because WGCNA was performed on a modest-sized bulk RNA-seq subset and no independent validation cohort was available, these findings should be interpreted as exploratory co-expression signatures rather than direct mechanistic evidence.

### 3.6. Prioritization and Expression Confirmation of Candidate Exercise-Modality-Associated Core Genes

**Figure 6:**
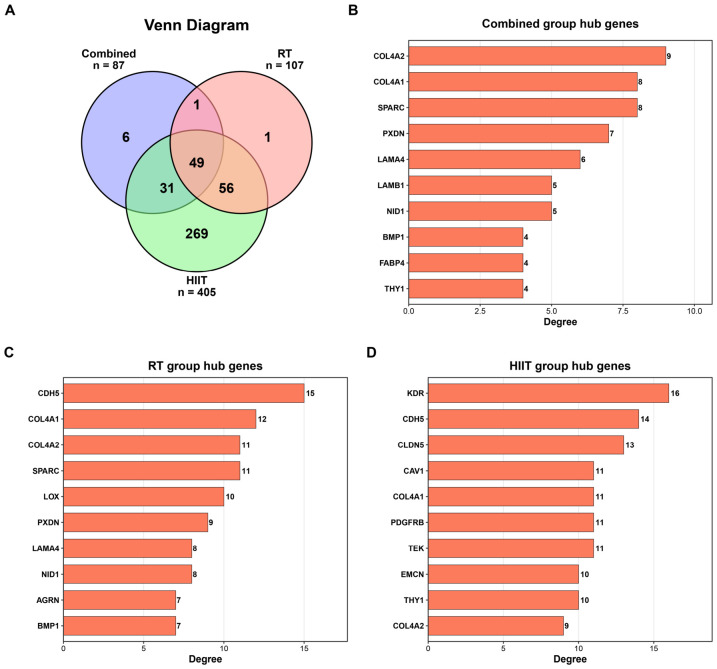
Candidate core gene analysis across exercise modalities. (**A**) Venn diagram showing the overlap of candidate genes among the combined, RT, and HIIT groups. Candidate genes were identified by intersecting DEGs with genes from exercise-responsive WGCNA modules. (**B**) Top ranked hub genes in the combined group PPI network. (**C**) Top ranked hub genes in the RT group PPI network. (**D**) Top ranked hub genes in the HIIT group PPI network. Hub genes were ranked by degree centrality. The x axis shows the degree. Dense PPI network plots are provided in Supplementary Figure S2.

To identify candidate core genes linked to exercise-responsive coexpression modules, we intersected genes from exercise-responsive WGCNA modules with DEGs from each exercise modality. This analysis identified 87 candidate genes in the combined group, 107 in the RT group, and 405 in the HIIT group (Figure 6A). Forty nine genes were shared across all three groups. HIIT contained the largest number of candidate genes, including 269 genes found only in the HIIT group. This pattern suggests a broader module-linked DEG profile after HIIT. However, this finding should not be interpreted as definitive evidence of stronger remodeling without formal testing between exercise modalities. We constructed PPI networks for each candidate gene set. To improve readability, dense PPI network plots were moved to Supplementary Figure S2. The main Figure 6 shows candidate gene overlap and the top ranked hub genes based on degree centrality. In the combined group, the top ranked hub genes included *COL4A2*, *COL4A1*, *SPARC*, *PXDN*, *LAMA4*, *LAMB1*, *NID1*, *BMP1*, *FABP4,* and *THY1* (Figure 6B). In the RT group, the top ranked hub genes included *CDH5*, *COL4A1*, *COL4A2*, *SPARC*, *LOX*, *PXDN*, *LAMA4*, *NID1*, *AGRN*, and *BMP1* (Figure 6C). In the HIIT group, the top ranked hub genes included *KDR*, *CDH5*, *CLDN5*, *CAV1*, *COL4A1*, *PDGFRB*, *TEK*, *EMCN*, *THY1,* and *COL4A2* (Figure 6D). These hub gene patterns suggest that combined training and RT were mainly linked to extracellular matrix and basement membrane remodeling, while HIIT showed a stronger vascular and endothelial pattern.

**Figure 7:**
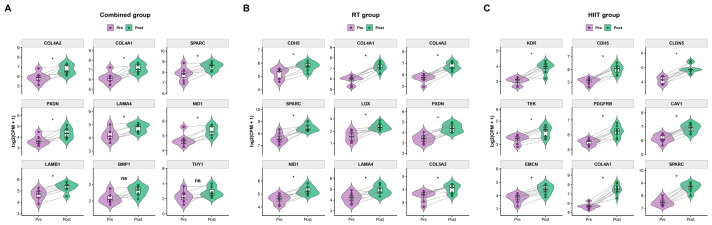
**Expression confirmation of selected candidate hub genes after exercise training.** (**A**) Paired expression levels of selected hub genes before and after training in the combined group. (**B**) Paired expression levels of selected hub genes before and after training in the RT group. (**C**) Paired expression levels of selected hub genes before and after training in the HIIT group. Expression values are shown as log2(CPM + 1). Lines connect paired samples from the same participant. Violin plots show expression distributions, and boxplots show the median and interquartile range. Purple indicates pre training samples, and green indicates post training samples. Statistical significance was assessed using paired Wilcoxon signed rank tests. * indicates *p* < 0.05; ns indicates not significant. Spearman correlation heatmaps for expression changes after training are provided in Supplementary Figure S3.

We next examined whether selected hub genes showed consistent expression changes after training. We compared normalized expression levels between paired samples collected before and after training. This analysis was used as expression confirmation, not as independent validation. The selected genes came from the same dataset through the DEG, WGCNA, and PPI workflow. In the combined group, most selected hub genes increased after training. *COL4A2*, *COL4A1*, *SPARC*, *PXDN*, *LAMA4*, *NID1*, and *LAMB1* were significantly upregulated (Figure 7A). *BMP1* and *THY1* did not reach statistical significance. In the RT group, selected hub genes, including *CDH5*, *COL4A1*, *COL4A2*, *SPARC*, *LOX*, *PXDN*, *NID1*, *LAMA4*, and *COL5A3*, increased after training (Figure 7B). In the HIIT group, vascular and endothelial-related genes, including *KDR*, *CDH5*, *CLDN5*, *TEK*, *PDGFRB*, *CAV1*, *EMCN*, *COL4A1*, and *SPARC*, were upregulated after training (Figure 7C). These results support the association of combined training and RT with extracellular-matrix-related signatures and the association of HIIT with vascular or endothelial transcriptomic signatures.

We also examined coexpression patterns among selected hub genes using Spearman correlation analysis. To improve readability, the correlation heatmaps were moved to Supplementary Figure S3. In the combined group, most selected genes showed positive correlations, especially extracellular matrix and basement membrane genes such as *COL4A2*, *COL4A1*, *SPARC*, *LAMA4*, *NID1*, and *LAMB1* (Figure S3A). In the RT group, most extracellular matrix and collagen-related genes were positively correlated (Figure S3B). CDH5 showed weaker correlations with several extracellular matrix genes. In the HIIT group, vascular and endothelial genes showed coordinated positive correlations, especially *KDR*, *CDH5*, *CLDN5*, *TEK*, and *PDGFRB* (Figure S3C). Together, these results support the relevance of these genes as candidate exercise-modality-associated network markers in aged skeletal muscle. Because these genes were identified through an exploratory DEG, WGCNA, and PPI workflow and examined within the same dataset, they should be interpreted as hypothesis-generating candidate genes rather than independently validated biomarkers or mechanistic drivers.

### 3.7. Regulatory Network Analysis Suggests Shared and Modality-Associated Candidate Regulators of Core Genes

To explore potential upstream regulators of selected hub genes, we constructed TF–mRNA and miRNA–mRNA regulatory networks using public database derived interactions. Hub genes identified through the DEG, WGCNA, and PPI workflow were used as input targets for each exercise modality. Regulators connected to these hub genes were extracted. The regulator degree was defined as the number of connected hub genes. Because all regulatory edges came from public databases, these results were interpreted as database-supported candidate regulatory relationships. They were not interpreted as direct evidence of TF or miRNA activity in the present samples.

**Figure 8:**
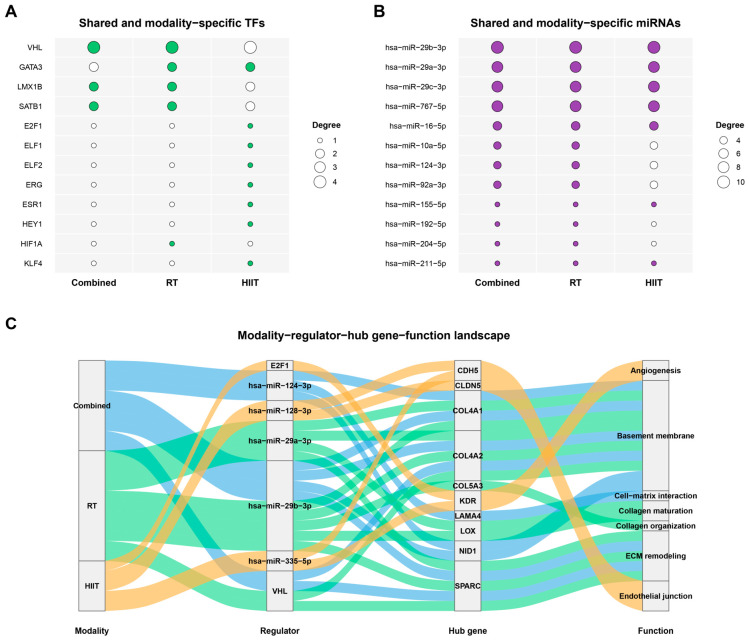
Regulatory landscape of selected candidate hub genes across exercise modalities. (**A**) Presence plot showing transcription factors connected with selected hub genes in the combined, RT, and HIIT groups. In panel A, green filled circles indicate the presence of TFs in the corresponding exercise modality, whereas open circles indicate absence. (**B**) Presence plot showing miRNAs connected with selected hub genes across the three exercise modalities. In panel B, purple filled circles indicate the presence of miRNAs in the corresponding exercise modality, whereas open circles indicate absence. The dot size indicates the regulator degree, defined as the number of connected hub genes. (**C**) Alluvial plot summarizing the relationships among the exercise modality, candidate regulator, hub gene, and functional category. In panel C, blue, green, and orange flows represent regulatory relationships associated with the Combined, RT, and HIIT groups, respectively. Regulatory relationships were derived from public database-based TF–mRNA and miRNA–mRNA interactions. Dense regulatory networks are provided in Supplementary Figure S4. These results were interpreted as database-supported candidate regulatory relationships rather than direct evidence of regulator activity.

The revised main Figure 8 summarizes the regulatory landscape in three panels. The TF presence plot showed shared and modality-related transcription factors across the combined, RT, and HIIT groups (Figure 8A). *VHL*, *LMX1B*, and *SATB1* were mainly observed in the combined and RT groups. Several TFs, including *E2F1*, *ELF1*, *ELF2*, *ERG*, *ESR1*, *HEY1*, and *KLF4*, were more evident in the HIIT group. The miRNA presence plot showed shared and modality-related miRNAs across the three exercise modalities (Figure 8B). Several miR-29 family members, including *hsa-miR-29a-3p*, *hsa-miR-29b-3p*, and *hsa-miR-29c-3p*, were present across all three groups. These miRNAs were linked to hub genes involved in extracellular matrix and vascular remodeling signatures.

The alluvial plot summarized the relationships among the exercise modality, regulator, hub gene, and functional category (Figure 8C). Combined training and RT were mainly linked to extracellular matrix, basement membrane, collagen organization, and ECM remodeling related genes, including *COL4A1*, *COL4A2*, *LAMA4*, *NID1*, *SPARC*, *LOX*, and *COL5A3*. HIIT was mainly linked to angiogenesis, endothelial junction, and vascular-remodeling-related genes, including *KDR*, *CDH5*, *CLDN5*, *TEK*, and *PDGFRB*. These results suggest that combined training and RT were more closely associated with ECM and collagen-related candidate regulatory patterns, whereas HIIT was more closely associated with vascular and endothelial candidate regulatory patterns.

To improve readability, the dense TF, miRNA, and hub gene regulatory networks were moved to Supplementary Figure S4. The combined network was mainly centered on extracellular-matrix- and basement-membrane-related hub genes. The RT network was mainly linked to collagen- and ECM-remodeling-related genes. The HIIT network showed a vascular and endothelial pattern. Overall, these findings suggest that different exercise modalities may be associated with distinct database-supported candidate regulatory landscapes. These findings should be interpreted cautiously because the regulatory relationships were derived from public databases. They do not establish direct regulator activity or causal regulatory mechanisms in the present dataset.

### 3.8. Transcriptome-Inferred Microenvironment Analysis Reveals Endothelial, Stromal, and Macrophage Signatures

We calculated marker-based microenvironment signature scores from bulk RNA-seq data to examine whether exercise-associated transcriptomic changes were linked to broader skeletal muscle microenvironment signatures. These signatures included endothelial, pericyte/mural, fibroblast/stromal, ECM/collagen, macrophage, T cell, B cell, satellite/myogenic, and adipocyte-related signatures. Because these scores were inferred from bulk RNA-seq profiles, they were interpreted as relative transcriptomic enrichment or activity signatures rather than direct measures of cell-type abundance.

**Figure 9:**
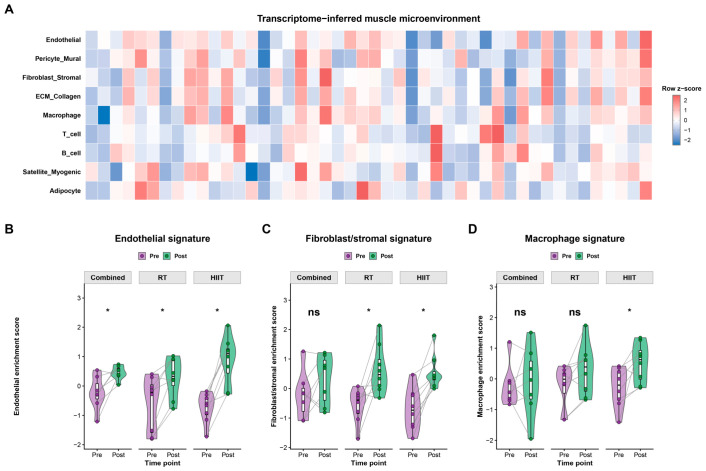
Transcriptome-inferred skeletal muscle microenvironment signatures after exercise training in older adults. (**A**) Heatmap showing transcriptome-inferred microenvironment signature scores across all samples. Rows represent endothelial, pericyte/mural, fibroblast/stromal, ECM/collagen, macrophage, T cell, B cell, satellite/myogenic, and adipocyte related signatures. Columns represent samples. Values are shown as row z scores. Sample labels on the x axis were hidden to reduce crowding. (**B**) Paired endothelial enrichment scores before and after training in the combined, RT, and HIIT groups. (**C**) Paired fibroblast/stromal enrichment scores before and after training across the three exercise modalities. (**D**) Paired macrophage enrichment scores before and after training across the three exercise modalities. Purple indicates pre-training samples, and green indicates post-training samples. Lines connect paired samples from the same participant. Paired Wilcoxon signed rank tests were used for statistical comparisons. * indicates *p* < 0.05; ns indicates not significant. The coupling analysis between microenvironment signatures and hub module scores, together with delta response analysis, is provided in Supplementary Figure S5. These scores were inferred from bulk RNA-seq marker expression. They should be interpreted as relative transcriptomic enrichment signatures rather than direct measures of cell type abundance.

The heatmap showed heterogeneous but structured microenvironment-related patterns across aged skeletal muscle samples (Figure 9A). The endothelial signature increased after training in the combined, RT, and HIIT groups (Figure 9B). The fibroblast/stromal signature increased after training in the RT and HIIT groups, whereas the combined group did not show a significant change (Figure 9C). The macrophage signature increased after training only in the HIIT group (Figure 9D). These results suggest that all three exercise modalities were associated with endothelial-related transcriptomic changes. RT and HIIT were also associated with fibroblast/stromal signatures, while HIIT showed an additional macrophage-related signature.

We also examined relationships between microenvironment signatures and module-level scores using post-training minus pre-training changes. Related microenvironment and hub module signatures showed positive associations, including endothelial with vascular hub, fibroblast/stromal with ECM hub, and macrophage with immune/inflammatory hub signatures. Subject-level delta-response analysis showed that HIIT had the largest mean increases in several signatures, including endothelial, vascular hub, ECM hub, macrophage, and immune/inflammatory scores (Table 1). However, no modality comparison remained significant after Benjamini–Hochberg FDR correction, so these findings were interpreted as exploratory trends. Overall, the results suggest coordinated endothelial, stromal/ECM, and immune-related transcriptomic signatures after exercise training in older adults, with HIIT showing a broader pattern. These findings should be interpreted cautiously because they were inferred from bulk RNA-seq marker expression and do not provide direct evidence of changes in cell-type abundance.

## 4. Discussion

This study reanalyzed skeletal muscle RNA-seq data from older adults in GSE97084. We compared transcriptomic responses after combined training, RT, and HIIT. The age-related loss of skeletal muscle function contributes to sarcopenia, poor physical performance, and metabolic dysfunction. Aged muscle also shows mitochondrial dysfunction, altered proteostasis, ECM remodeling, and changes in the tissue microenvironment [11,14]. Our results suggest that exercise training was associated with both shared and modality-related transcriptomic signatures in aged human skeletal muscle. Combined training and RT were mainly linked to ECM, collagen, basement membrane, adhesion, and stromal remodeling. HIIT showed broader vascular endothelial, angiogenic, mitochondrial, immune-related, and microenvironment-related signatures.

The broader DEG profile after HIIT is consistent with the original report by Robinson et al., which showed strong transcriptomic and mitochondrial adaptations after HIIT in older adults [8]. However, this result does not mean that HIIT is biologically superior to the other exercise modalities. It only suggests a broader transcriptional response under the current analysis. This interpretation is consistent with the view that exercise adaptation involves coordinated changes across multiple biological layers [23,24]. Previous studies have also linked HIIT to improved endothelial function through shear stress, nitric oxide signaling, oxidative stress regulation, and inflammatory modulation [25].

WGCNA supported these findings at the module level. This method identifies groups of genes with coordinated expression and complements single-gene analysis [18]. In this study, MEturquoise showed the strongest association with HIIT. MEsalmon showed negative changes across exercise modalities. These results suggest that exercise training may affect coordinated gene programs in aged skeletal muscle. The subject level delta-response analysis also suggested larger mean vascular and microenvironment responses after HIIT. However, these effects did not remain significant after Benjamini–Hochberg FDR correction. Therefore, these findings should be interpreted as exploratory trends rather than confirmed differences among exercise modalities. The WGCNA results also require validation because the analysis used a modest-sized bulk RNA-seq subset.

ECM and collagen remodeling were consistent features of the combined and RT groups. These signals appeared in the enrichment analysis, GSEA, PPI analysis, hub gene expression patterns, and WGCNA. Representative genes included *COL4A1*, *COL4A2*, *SPARC*, *PXDN*, *LAMA4*, *NID1*, *LAMB1*, *LOX*, and *COL5A3*. These genes are related to basement membrane organization, collagen-containing ECM, matrix assembly, and ECM maturation. This pattern is biologically plausible because the skeletal muscle ECM supports force transmission, tissue repair, satellite cell regulation, fibrosis, and muscle remodeling [11]. Exercise training can also alter ECM-related transcripts and proteins in locomotor muscles [12]. The ECM pattern after RT may reflect the mechanical loading nature of resistance exercise. Mechanical force can act through the ECM, integrins, and the cytoskeleton to support structural adaptation [26]. *LOX* was also repeatedly identified in RT-associated networks. This finding is relevant because *LOX*-family enzymes contribute to collagen and elastin crosslinking and matrix stability [27]. However, this study measured mRNA expression. The data do not directly show ECM protein deposition, collagen crosslinking, or tissue stiffness.

HIIT showed a clearer vascular endothelial signature. Hub genes related to HIIT included *KDR*, *CDH5*, *CLDN5*, *TEK*, *PDGFRB*, *CAV1*, and *EMCN*. These genes are involved in angiogenesis, endothelial junctions, vascular barrier function, pericyte interactions, and vessel stability [28,29,30,31,32]. This pattern is relevant to aging muscle because aging can impair angiogenesis and alter ECM and vascular interactions [14]. The ECM composition, matrix stiffness, and basement membrane structure can regulate endothelial migration, sprouting, lumen formation, and vascular stabilization [33,34]. HIIT may provide strong metabolic and hemodynamic stimuli, including an increased oxygen demand, energetic stress, and shear stress. Exercise-induced skeletal muscle angiogenesis is also regulated by VEGF signaling, shear stress, nitric oxide signaling, and basement membrane remodeling [13]. The microenvironment analysis supported this vascular pattern. Endothelial signatures increased after all three exercise modalities. Fibroblast and stromal signatures increased after RT and HIIT. Macrophage signatures increased after HIIT. These scores were inferred from bulk RNA-seq data. They should be interpreted as relative transcriptomic enrichment signatures rather than direct measures of cell type abundance [22,35,36]. The macrophage signal after HIIT may reflect immune remodeling or tissue-repair-related activity. Macrophages contribute to muscle repair, regeneration, inflammation resolution, and interactions with stromal and satellite cells [37,38,39]. The present data cannot determine whether this signal reflects macrophage abundance, the activation state, or macrophage and stromal interaction.

The regulatory network analysis suggested possible upstream regulatory patterns. In the combined and RT groups, miR-29 family members were repeatedly connected with ECM related hub genes, including *COL4A1*, *COL4A2*, *SPARC*, and *LOX*. This finding is consistent with previous evidence that the miR-29 family regulates ECM- and fibrosis-related genes [40,41]. In the HIIT group, vascular endothelial hub genes were connected with a broader set of candidate TFs and miRNAs. This pattern suggests possible regulatory relationships related to angiogenesis, endothelial junctions, and vascular remodeling. However, these relationships were derived from public databases through NetworkAnalyst, TRRUST, and miRTarBase v9.0 [19,20,21]. They should be viewed as database-supported candidate relationships. They do not prove TF or miRNA activity in the present samples.

This study has several strengths. The paired design before and after training reduced the influence of baseline differences between participants. The integrated workflow combined differential expression, pathway analysis, WGCNA, PPI analysis, regulatory network inference, microenvironment signature analysis, and a subject-level delta response analysis. This approach provided a broader view than differential expression analysis alone. This study also has limitations. It used a single public bulk RNA-seq dataset with a modest number of older participants. It lacked an independent validation cohort and did not include a younger reference group. Most analyses were based on paired comparisons within each exercise modality. These analyses did not provide strong evidence supported by FDR for gene-level time by modality interactions. WGCNA modules, PPI networks, regulatory networks, and microenvironment scores also need cautious interpretation. Future studies should test these findings in larger cohorts and with complementary methods, such as proteomics, histology, immunostaining, single cell RNA-seq, spatial transcriptomics, and direct physiological measurements.

Overall, this reanalysis suggests that exercise training may remodel aged human skeletal muscle through both shared and modality-related transcriptomic programs. Combined training and RT were mainly associated with ECM and structural remodeling signatures. HIIT showed broader vascular endothelial, immune-related, metabolic, and microenvironment signatures.

## 5. Conclusions

This exploratory reanalysis suggests that combined training, RT, and HIIT were associated with partly overlapping but distinct transcriptomic signatures in aged human skeletal muscle. Combined training and RT were mainly linked to ECM, collagen, adhesion, basement membrane, and stromal remodeling signatures. HIIT showed a broader pattern that included vascular endothelial, angiogenic, mitochondrial, immune-related, and microenvironment-related signatures.

These findings support the view that exercise may remodel aged skeletal muscle through coordinated changes in myofibers, the extracellular matrix, the vascular endothelium, stromal components, and immune-related signals. These results should be interpreted as hypothesis-generating because this study used a modest older adult subset from a single public bulk RNA-seq dataset and lacked independent validation. Larger independent cohorts and complementary experimental studies are needed to confirm these modality-related transcriptomic patterns.

## Figures and Tables

**Table 1:** Subject-level delta-response comparisons across exercise modalities.

Signature Category	Delta-Response Signature	Combined Δ Mean ± SEM	RT Δ Mean ± SEM	HIIT Δ Mean ± SEM	Omnibus *p* Value	FDR-Adjusted *p* Value
Microenvironment signature	Endothelial-related score	0.685 ± 0.218	0.905 ± 0.226	1.625 ± 0.239	0.023	0.068
Module-level signature	Vascular hub score	0.778 ± 0.263	1.052 ± 0.187	1.776 ± 0.261	0.021	0.068
Module-level signature	ECM hub score	1.042 ± 0.268	1.285 ± 0.222	1.891 ± 0.203	0.046	0.092
Microenvironment signature	Fibroblast/stromal-related score	0.534 ± 0.364	1.220 ± 0.366	1.258 ± 0.209	0.234	0.281
Microenvironment signature	Macrophage-related score	0.139 ± 0.423	0.533 ± 0.212	0.811 ± 0.309	0.353	0.353
Module-level signature	Immune-inflammatory score	0.059 ± 0.306	0.211 ± 0.262	0.787 ± 0.268	0.172	0.258

Note: values are shown as the mean ± SEM and represent post-training minus pre-training delta-response scores. Each row represents one microenvironmental or module-level signature. Omnibus *p* values were calculated using Kruskal–Wallis tests across the combined, RT, and HIIT groups. FDR-adjusted *p* values were calculated using the Benjamini–Hochberg method across all tested signatures. No comparison remained significant after FDR correction. Therefore, nominal *p* values were interpreted only as exploratory trends rather than statistically significant findings.

## Data Availability

The raw RNA-seq data analyzed in this study are publicly available from the Gene Expression Omnibus database under accession number GSE97084. The processed data, differential expression results, enrichment results, WGCNA results, and analysis scripts supporting the findings of this study are provided as Supplemental Files.

## Supplementary Materials

The following supporting information can be downloaded at: https://www.mdpi.com/article/10.3390/genes17070803/s1. Figure S1: DEG number comparison and DEG overlap across exercise modalities; Figure S2: Dense PPI networks for candidate genes across exercise modalities; Figure S3: Spearman correlation heatmaps of selected hub gene expression changes after training; Figure S4: Dense TF–mRNA and miRNA–mRNA regulatory networks across exercise modalities; Figure S5: Coupling analysis between transcriptome-inferred microenvironment signatures and hub module scores, together with subject-level delta-response analysis.
