# Supplementary figures and images for: Transcriptomic Signatures of Exercise-Modality Responses in Aged Human Skeletal Muscle
Source: Genes (Basel). 2026 Jul 15;17(7):803. doi: 10.3390/genes17070803 (PMC13409252; doi:10.3390/genes17070803)

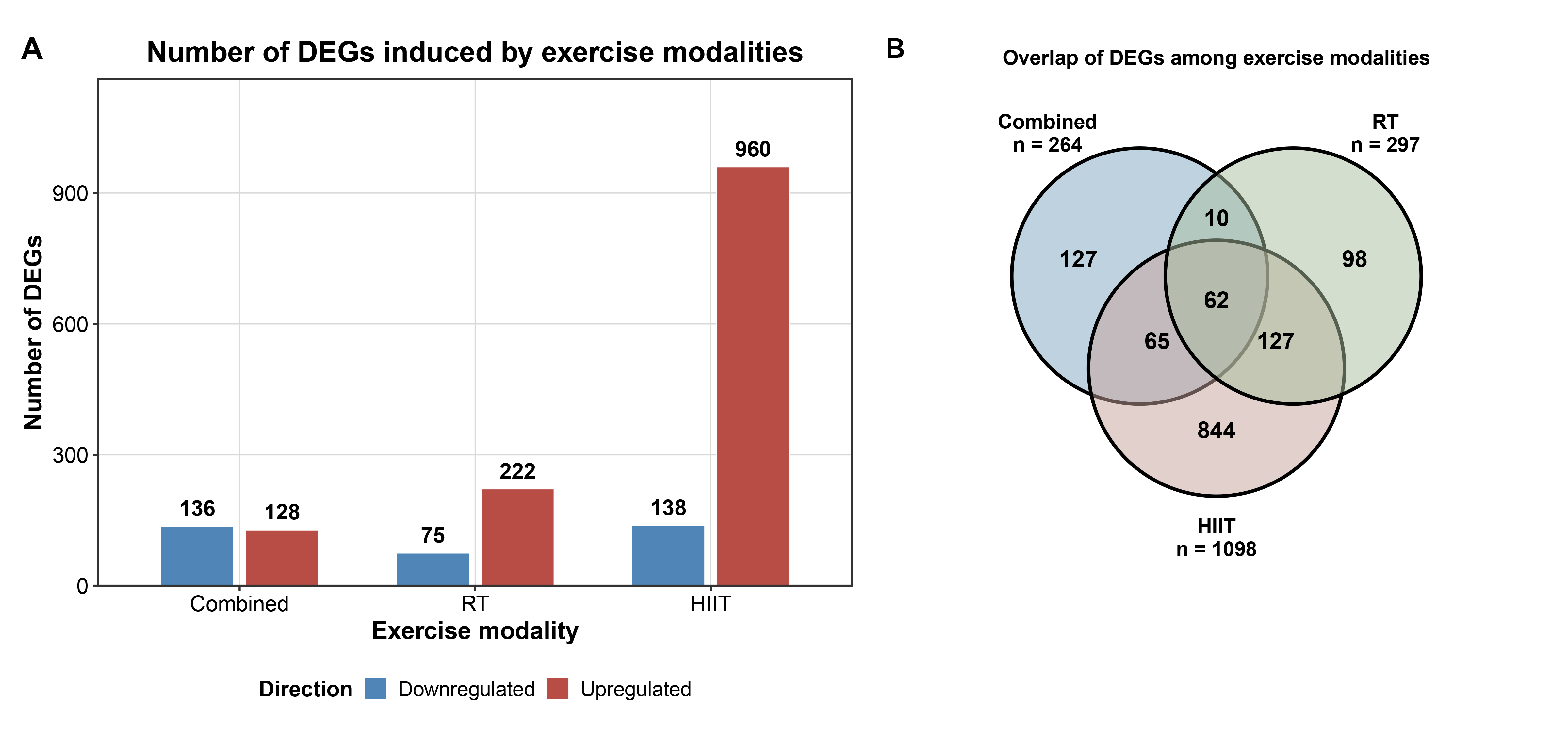

Supplement: Supplementary file 1 [file genes-17-00803-s001.zip › Supplementary_Figure_S1.tif]

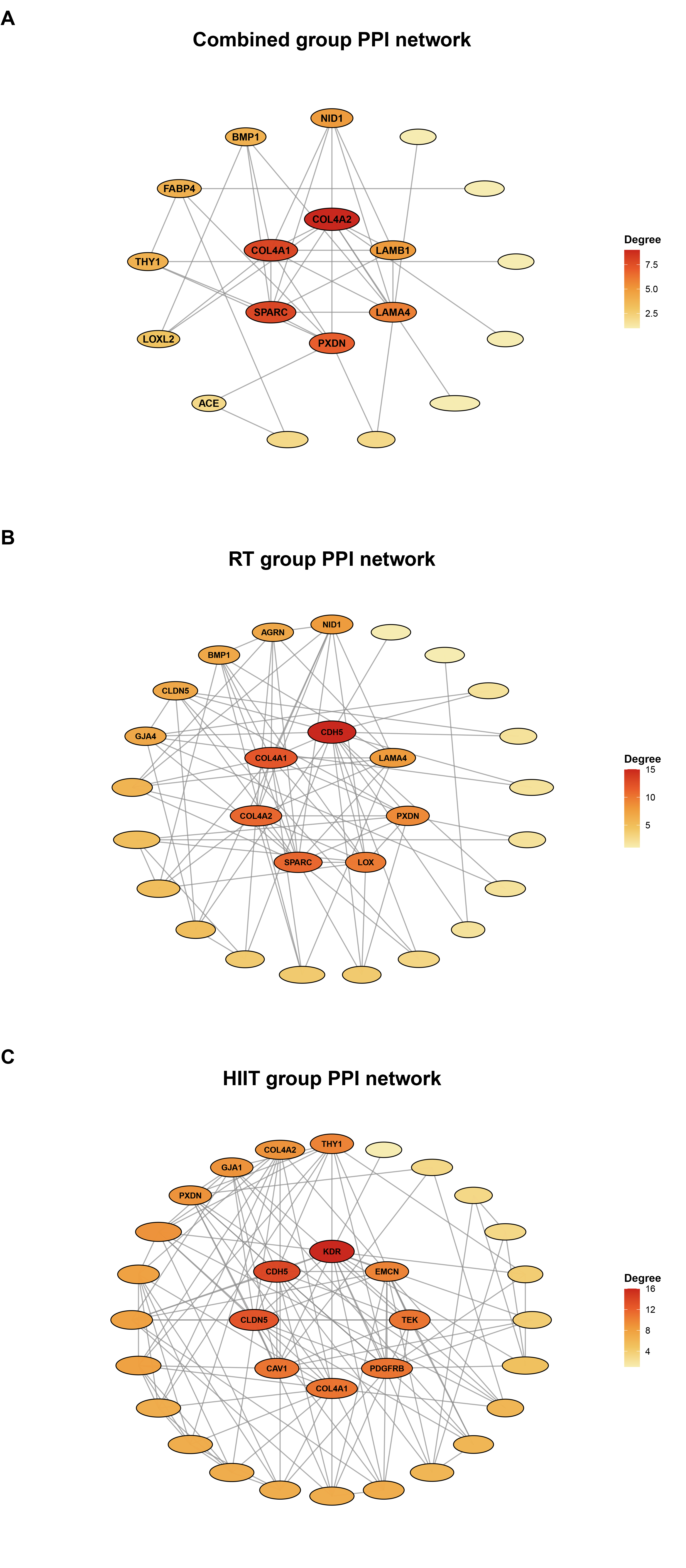

Supplement: Supplementary file 1 [file genes-17-00803-s001.zip › Supplementary_Figure_S2.tif]

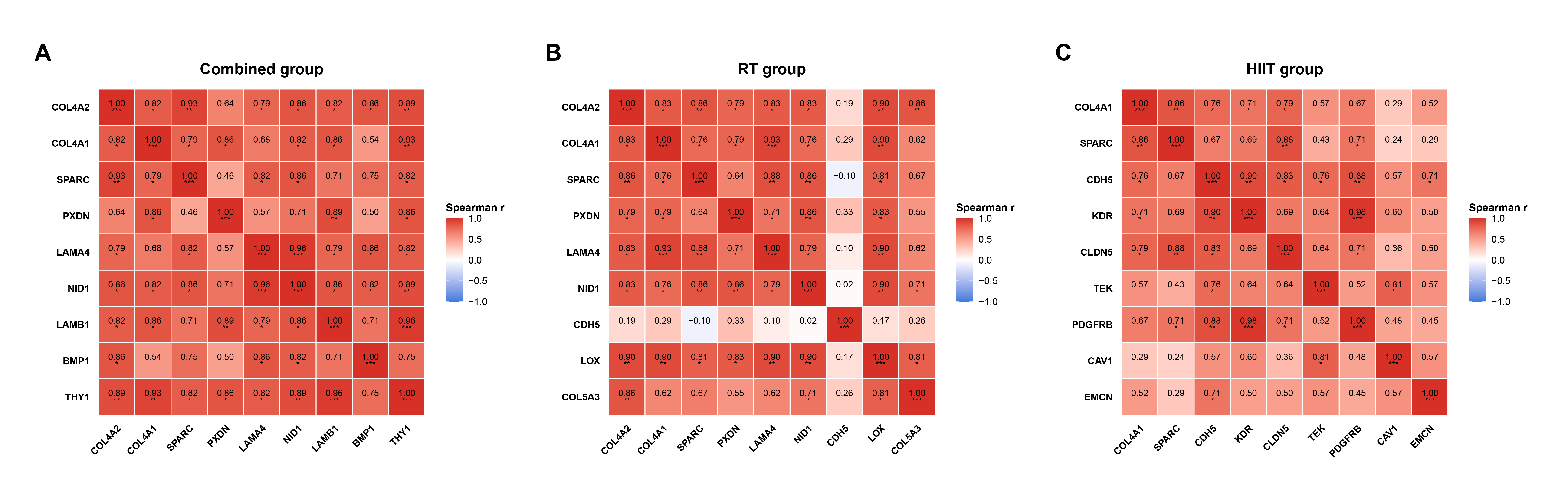

Supplement: Supplementary file 1 [file genes-17-00803-s001.zip › Supplementary_Figure_S3.tif]

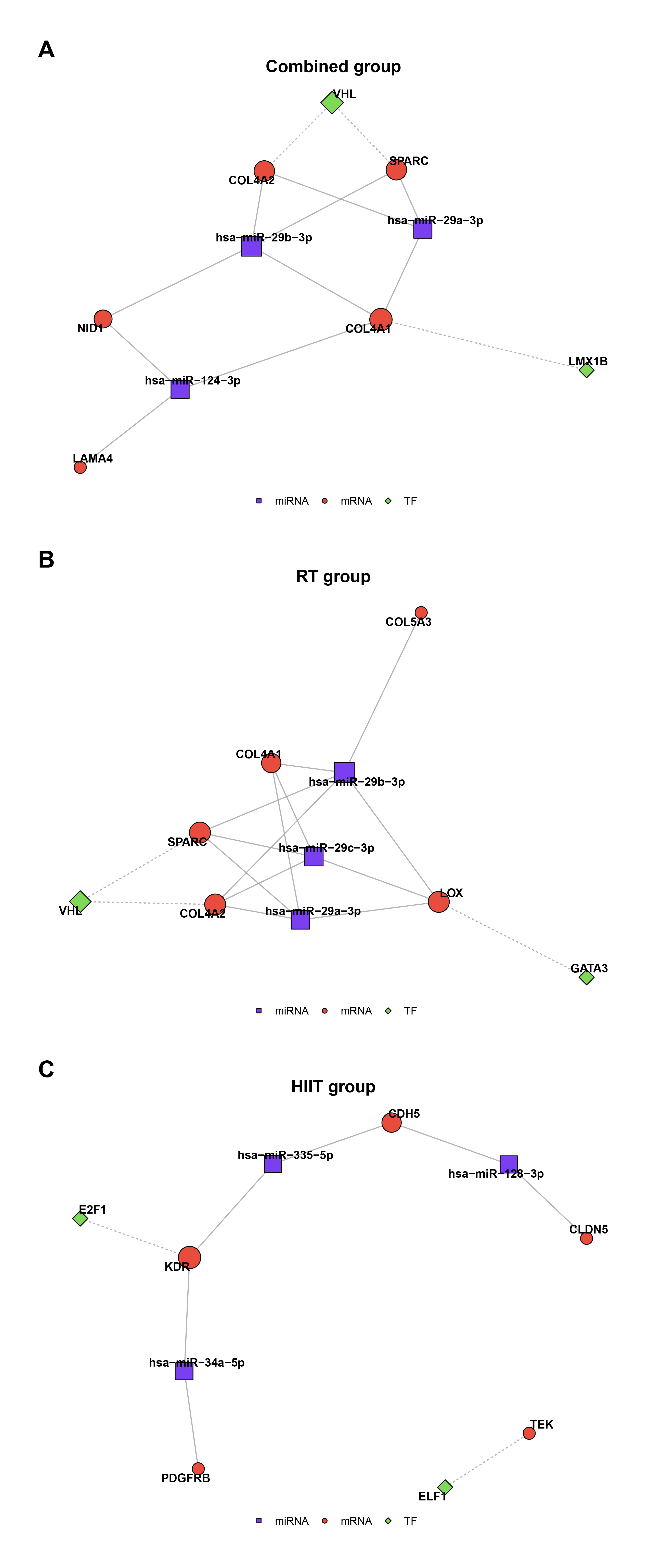

Supplement: Supplementary file 1 [file genes-17-00803-s001.zip › Supplementary_Figure_S4.tif]

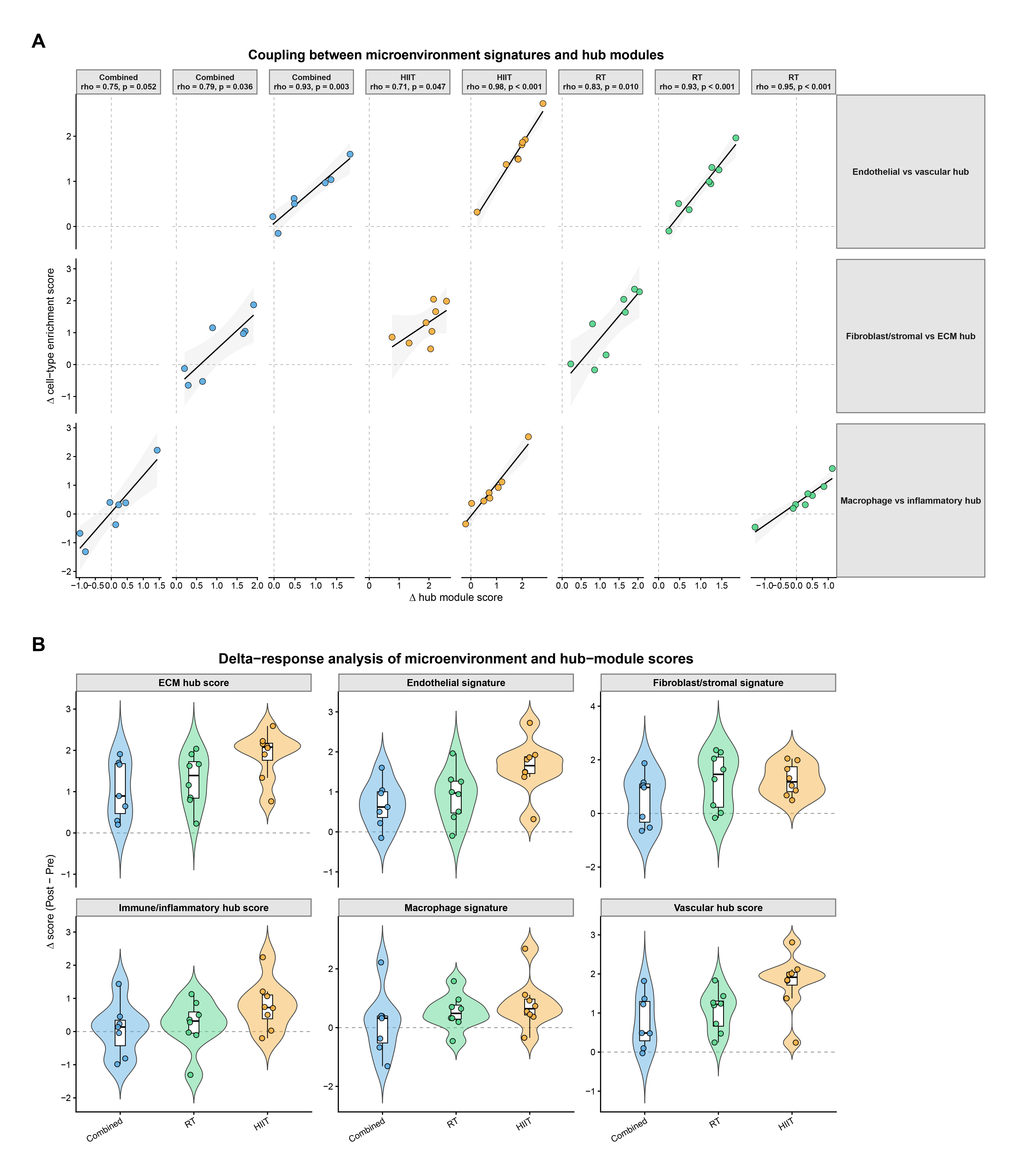

Supplement: Supplementary file 1 [file genes-17-00803-s001.zip › Supplementary_Figure_S5.tif]
